# Prevalence and associated factors of postpartum depression among immigrant women in Guangzhou, China

**DOI:** 10.1186/s12884-020-02946-4

**Published:** 2020-04-25

**Authors:** Ribo Xiong, Aiwen Deng

**Affiliations:** 1 Department of rehabilitation, Nanhai Hospital, Southern Medical University, Foshan, China; 2 General Practice Center, Nanhai Hospital, Southern Medical University, Foshan, China

**Keywords:** Immigrant, Postpartum depression, Prevalence, Associated factors, Social support

## Abstract

**Background:**

Although there has been mounting research on postpartum depression (PPD), the impact of immigration on PPD has remained quite unexplored. The purpose of this study is to investigate the prevalence and associated factors of PPD among immigrant women living in Guangzhou at 6 weeks postpartum.

**Methods:**

A cross-sectional study was conducted involving 1230 immigrant women in a tertiary hospital of Guangzhou from December 2016 to December 2017 at 6 weeks postpartum. The Chinese version of Edinburgh Postnatal Depression Scale and a structured questionnaire regarding associated factors were administered to all participants. Multivariate logistic regression was used to determine factors that were significantly associated with PPD.

**Results:**

The prevalence of PPD among immigrant women at 6 weeks postpartum was 34.0%. A multivariate logistic regression model identified significant obstetric and social factors as: living in Guangzhou for less than 2 years, insufficient family income, poor social support and marital relationship**.**

**Conclusion:**

Prevalence of PPD among immigrant women from Guangzhou at 6 weeks postpartum is high. The development of PPD among immigrant women is associated with individual and social factors. There’s an urgent need for healthcare providers to take a more active role in engaging immigrant women in their psychological needs.

## Background

Over the course of the last two decades or so, China has experienced the most extensive internal migration which has brought increased attention to the promotion of migrant-sensitive health policies and diseases prevention especially for migrant women and children. According to national statistics, up to 2015, an estimated 277 million immigrants who were originally from the poor and rural areas in western and central inland provinces migrated to southern and eastern developed regions, such as Shanghai and Guangzhou for better opportunities and income [1]. Based on the definition of immigrants in the National Internal Migrant Dynamic Monitoring Survey, immigrants in our study refers to the individuals who do not have permanent resident certificate(“Hukou”) and should be living over a month in the residence. Due to the fact that the utility and allocation of public resources is based on household registration (“Hukou”) policy, immigrants do not have the same rights and benefits as local registered residents in a variety of areas, such as health care, social services, offspring education and housing. In addition, immigrants have confronted challenges in a new host society, such as adjusting to a new language, unfamiliar laws, different norms for social interaction and lifestyle changes. How the acculturation affects immigrants’ health status and the relationship between immigration process and the mental health of women continuously attract researcher’s interest.

A growing number of studies suggest that immigrant women may be at increased risk of depression prenatally and postnatally [2–4]. A recent systematic review found that immigrant women are twice as likely to experience PPD as nonimmigrant women [5]. In a cross-sectional study conducted in Tokyo, Japan, migrant Chinese women had elevated depression symptoms postnatally than Japanese-born women [6]. Several studies conducted in Ontario, Canada have found migrant Chinese women had increased risk of PPD compared to their Canadian-born counterparts [7, 8]. The existing literature mainly focused on Chinese migrants in Canada and other developed countries with significant disparities in culture and social environment. The status of PPD among Chinese internal migrants have remained quite unexplored. This is a significant limitation given the rapidly changing demographics in the south area of China where about 1 in 3 are identified as immigrants.

The principle aim of the present study is to examine the occurrence of PPD at 6 weeks after delivery among immigrant women. By exploring the biological, psychological and cultural factors, culturally appropriate and equitable health care services are provided to support immigrant mothers.

## Methods

### Participants

This is a cross-sectional study carried out from December 2016 to 2017 in a tertiary hospital of Guangzhou.The effect size was assumed to be 0.30, referring the study of Gaskin, et al. [9] To achieve 80% power with an effect size of 0.5 and an alpha of 0.05, a total sample size of 367 was needed. We recruited female immigrants at 6 weeks after childbirth 1) at childbearing age between 18 and 49;2) without “Hukou”(registered resident certificate) in Guangzhou and be living over a month in Guangzhou; 3) Chinese nationality; 4)providing informed consent. Women who had a history or family history of psychiatric diseases were excluded. Women who returned incomplete questionnaires were also excluded. A total of 1247 eligible women were invited to participate; 5 women refused to be enrolled in and 12 women returned incomplete questionnaires, resulting 1230 women. This study included 1230 immigrant women, allowing detection of significant differences with a power of 0.91 calculated by GPower software.

### Procedures and measures

Investigators conducted face to face interview for data collection. The Chinese version of Edinburgh Postnatal Depression Scale (EPDS) was employed to assess PPD with a threshold of 10. The sensitivity and specificity of Chinese version have been found to be 0.82 and 0.86 respectively which were comparable to the original scale [10].

Based on literature [11]and expert consultation, structured questionnaires [12] were administered to each women covering information including: 1) socio-demographic factors such as age, employment, labor contract, annual household income, years of residence; 2) obstetric factors such as parity, planned pregnancy, mode of delivery, pregnancy associated diseases, delivery associated diseases and fetus gender; 3) social factors such as perceived family income sufficiency, family socio-economic status, the ability to use local language, social support and marital relationship. Social support was ascertained by Likert scale with a range from 12 to 60. The scale consists of three subscales: informational support (i.e, provision of advice, suggestions, information that the women could use), emotional support (i.e, provision of empathy, love, and caring), and household activity support. This scale demonstrated acceptable reliability and validity in a previous study [13].

The study was approved by the Research Ethics Board of Southern Medical University and written consent was sought from eligible participants.

### Data analysis

The primary data was entered into Epidata3.0 before being exported to SPSS16.0. Chi-square test was used to assess the differences between two independent qualitative data and Student’s *t* test was used for quantitative data. Then the multivariate logistic regression was further employed to identify factors that were significantly associated with PPD. A *P* value <0.05 was considered significant in the analysis.

## Results

Out of 1230 immigrant women who were enrolled in the assessment, we have reported that 418 women screened positive for PPD with EPDS at a cut-off point of 10, thus resulting in a prevalence of 34.0%.

Analysis of demographic profiles of immigrant women was shown in Table 1. Among all the factors concerning age, employment, labor contract, annual household income and years of residence, less recent immigrants showed significantly lower rates of PPD compared with recent immigrants.

**Table 1:** Demographic profiles of immigrant women with PPD

Factors	EPDS <10 (812)		EPDS ≥10 (418)		***χ***^***2***^	***P***
	n	%	n	%		
Age					1.010	0.799
≤ 24	123	15.15	67	16.03		
25 ~ 29	504	62.07	260	62.20		
30 ~ 34	112	13.79	60	14.35		
≥ 35	73	8.99	31	7.42		
Employment					0.516	0.259
Yes	553	68.10	293	70.10		
No	259	31.90	125	29.90		
Labor contract					2.275	0.810
Unfixed term	298	36.70	160	38.28		
Fixed term	275	33.87	142	33.97		
One-time task or a probation period	138	17.00	69	16.50		
Unsign labor contract	79	9.73	35	8.37		
Unknown	14	1.72	10	2.39		
Else	8	0.98	2	0.48		
Annual household income					5.164	0.160
60,000 ~ 99999RMB	210	25.86	130	31.10		
100,000 ~ 139999RMB	389	47.90	187	44.74		
140,000 ~ 179,999 RMB	131	16.13	55	13.16		
≥ 180000RMB	82	10.10	46	11.00		
Years of residence					74.383	0.000
≤ 2 years	190	23.40	188	44.98		
2 ~ 5 years	324	39.90	155	37.08		
6–10 years	298	36.70	75	17.94		

Analysis of obstetric factors and probable PPD was shown in Table 2. There were no significant differences between immigrant women with PPD and those without regarding obstetrics factors.

**Table 2:** Obstetric characteristics of immigrant women with PPD

Factors	EPDS <10 (812)		EPDS ≥10 (418)		***χ***^***2***^	***P***
	n	%	n	%		
Parity					0.159	0.706
1	291	36.21	145	34.69		
≥ 2	521	64.16	273	65.31		
Pregnancy planning					1.889	0.184
Unplanned	423	52.09	235	56.22		
Planned	389	47.91	183	43.78		
Mode of delivery					1.736	0.207
Vaginal	624	76.85	307	73.44		
Caesarean	188	23.15	111	26.56		
Pregnancy related disease					0.266	0.632
Yes	217	26.72	106	23.36		
No	595	73.28	312	74.64		
Delivery related disease					0.723	0.434
Yes	108	13.30	63	15.07		
No	704	86.70	355	84.92		
Fetus gender					0.724	0.399
Male	428	52.71	231	55.26		
Female	384	47.29	187	44.74		

Table 3 represented social factors and probable PPD. Insufficient family income, lower social support and poor marital relationship were significantly associated with the development of PPD among immigrant women.

**Table 3:** Social profiles of immigrant women with PPD

Factors	EPDS <10 (812)		EPDS ≥10 (418)		***χ***^***2***^***/t***	***P***
	n %		n	%		
Perceived family income sufficiency					95.088	0.000
Not enough	259	31.90	251	60.05		
Just enough	408	50.25	139	33.25		
More than enough	145	17.85	28	6.70		
Family socio-economic status					3.102	0.212
Low	263	32.39	147	35.17		
Middle	514	63.30	246	58.85		
High	35	4.31	25	5.98		
The ability to use local language					2.878	0.237
Poor	468	57.64	259	61.96		
Moderate	240	29.56	117	27.99		
Satisfying	104	12.80	42	10.05		
Social support	38.42 ± 9.21		31.08 ± 10.24		−15.655	0.000
Marital relationship					104.2	0.000
Poor	178	21.92	210	50.24		
Moderate	245	30.17	92	22.01		
Satisfying	389	47.91	116	27.75		

Table 4 showed the associated factors of PPD in immigrant women. The strongest factor was lower social support. Immigrant women with lower social support was 3.287 times more likely to develop PPD. In relation to perceived family income, immigrant women who reported insufficiency of family income were at greater risk of PPD (OR = 2.413, 95%CI: 1.991 ~ 3.134). Similarly, poor marital relationship was identified as a risk factor (OR = 2.317, 95%CI: 1.917 ~ 2.739). An increased risk was also observed in immigrant women with less than 2 years of residence in Guangzhou (OR = 3.018, 95%CI: 2.589 ~ 3.422).

**Table 4:** Multivariate logistic regression analysis of impact factor of PPD in immigrant women

Covariates	***B***	***S.E***	***Wald***	***P***	***OR***	***95%CI***
Age	0.087	0.151	14.219	0.658	1.041	0.455 ~ 1.328
Employment	0.973	0.824	28.982	0.471	2.634	2.006 ~ 3.140
Labor contract	0.056	0.138	17.231	1.025	3.177	2.216 ~ 3.841
Annual household income	0.991	1.472	15.365	3.007	1.945	1.090 ~ 3.149
Years of residence						
≤ 2 years	1.763	0.381	33.105	0.000	3.018	2.589 ~ 3.422
2 ~ 5 years	−0.897	0.153	27.314	0.094	1.229	0.877 ~ 1.681
6–10 years	−0.776	0.169	19.233	1.241	1.586	1.040 ~ 1.989
Parity	1.263	4.287	47.116	0.987	2.019	1.114 ~ 2.840
Pregnancy planning	0.016	0.125	3.684	0.065	1.217	0.830 ~ 1.607
Mode of delivery	−0.339	0.700	56.139	1.162	2.311	2.059 ~ 3.327
Pregnancy related disease	0.159	0.230	9.116	3.670	1.652	1.162 ~ 2.080
Delivery related disease	0.094	0.113	14.214	0.951	1.643	0.744 ~ 2.016
Fetus gender	0.052	0.136	10.050	0.083	2.097	1.339 ~ 3.017
Perceived family income sufficiency						
Not enough	0.631	1.220	15.687	0.000	2.413	1.991 ~ 3.134
Just enough	0.248	0.746	11.501	0.077	1.359	0.423 ~ 2.982
More than enough	–	–	–	–	–	–
Family socio-economic status	−0.467	0.189	17.564	0.136	1.153	0.671 ~ 1.622
The ability to use local language	−0.990	0.231	56.071	0.068	2.376	2.119 ~ 3.075
Social support	1.842	0.126	32.005	0.000	3.287	2.903 ~ 3.720
Marital relationship						
Poor	0.021	0.108	44.168	0.000	2.317	1.917 ~ 2.739
Moderate	0.069	0.504	34.180	0.106	1.288	0.636 ~ 1.751
Satisfying	–	–	–	–	–	–

Adjusted for all the above variates

## Discussion

Guangzhou, where this study was conducted, is one of the largest migrant-concentrated metropolis in China. With nearly 50% of it’s total population being migrated and 50% of it’s migrants being female, Guangzhou provided a natural environment where the maladjustment such as depression and anxiety can be observed among immigrant women [14]. The prevalence rate of PPD was as high as 34.0%. Likewise, a Japanese study of new mothers found that 54.5% of migrant Chinese women had elevated levels of postpartum depressive symptoms as indicated by scores on EPDS [6]. Several other Canadian studies similarly found that the risks for PPD in immigrant Chinese women were significantly higher than that of native-born women [2–4]. In another sample of immigrant women in Taiwan, higher prevalence of PPD (41.1% vs. 8.4%) was also identified [15]. However, studies that compare US-born Hispanic mothers with their foreign-born counterparts suggested a lower rate in the latter [16]. In another epidemiological study, no difference of PPD rates was observed among foreign-born mothers and US-born mothers, but not among Whites or Asian/Pacific Islanders [17]. The variation of the prevalence highlights the complex interplay of social, cultural and systemic factors in the development of PPD. Higher rates of PPD in this study may be explained by differences across time point, social and cultural norms. First, women were screened for PPD at 6 weeks postpartum. Generally speaking, the rates obtained closer to delivery are more likely to be higher than those conducted later because of the care and support from their families after childbirth [18]. Second, China’s internal migrants encountered “Hukou”-based social exclusion rather than ethnicity-based social exclusion experienced by international migrants. They are also more likely to face barriers such as knowledge regarding access to services and differences in language and lifestyle. We hypothesized social exclusion resulting from the “Hukou” policy as well as other economic and cultural factors are associated with adverse mental health consequences of immigrants. Further study is needed to explore the relationship between social exclusion and PPD among immigrant women.

This is one of the few studies examining length of time in the host society as an associated factor of PPD at 6 weeks. Having lived in Guangzhou for less than 2 years was significantly associated with PPD at 6 weeks. Similarly, immigrant women within previous 2 years in Canada were at increased risk for PPD at 6 weeks postpartum [11]. This might be attributed to the difficulties in adaptation to the host society and absence of usual support that would have been received from their family of origin. Furthermore, newcomers may have practical barriers such as equitable access to postnatal care, language difficulties and fear of stigma. This result has high policy relevance for local government to promote social integration for immigrants.

We also found a strong association between perceived family income insufficiency and PPD at 6 weeks postpartum. Our finding of perceived family income as an important factor of PPD is consistent with previous reviews focused on the overall postpartum population and a recent research on determinants of PPD among Chinese immigrants to high-income countries [5, 11, 19]. It’s noteworthy that immigrant women were at higher risk of PPD even after accounting for insufficient family income, social support and domestic making power [20]. One of the possible explannation could be that immigrant mothers are mostly financially dependent on their spouses. The decreased status in the family as well as the stress of adapting to a new society could lead to a sense of helpless. This in turn could be related to less domestic making power which has been demonstrated to be a predictor of PPD in a research [20]. Future study could include women from different cultural backgrounds to better understand the relationship between family income and PPD among immigrant mothers.

Low social support was coherently associated with the development of PPD and has greater implications for immigrant women with limited or no social support [11, 18]. For the immigrant women in our study, those who received lower social support were up to three times more likely to develop PPD. It’s a cultural imperative for Chinese women to observe a special tradition after childbirth called “doing the month”. This tradition includes a set of practices involving prescribed diet, clothing and other cautions that must be followed during the first month after delivery. Numerous studies have revealed “doing the month” have positive influence on the recovery of the mother and successful adaptation to maternal role [21, 22]. Not following this ritual could lead to postpartum difficulties. However, because of comparatively lower income, unstable housing and absence of caregivers during the period of “doing the month”, the psychosomatic recovery of immigrant mothers are negatively affected. The impact of low social support on PPD highlights the need to improve social welfare system for immigrants.

Poor marital relationship played an important role in the occurrence of PPD as observed in previous studies [5]. A research revealed feeling of isolation and loneliness resulting from poor marital relationship may lead to depressive symptoms among Thai women living in Australia [18]. Since most immigrant mothers depend on their husband financially, it’s possible that financial challenges or lack of social networks are associated with increased prevalence of PPD. On the other hand, in Chinese culture, poor marital relationship or even domestic violence is regarded as stigma. Immigrant women may be reluctant to seek help in order to maintain the idealization of motherhood as a joyful period.

The results of our study should be considered with a few caveats. Firstly, a cross-sectional study did not allow us to establish a causal relationship. Secondly, EPDS was employed as screening tool to define PPD instead of a clinician-administered structured diagnostic interview. Thirdly, the recruitment of women from one of the districts in Guangzhou would limit the generalizability of the findings. Fourthly, report bias may exist regarding parity and sufficiency of family income. Given the sensibility of the topics, migrants might feel shy to reveal or decline because of social desirability bias.

## Conclusions

The prevalence rate of PPD among immigrant women was high in Guangzhou, China. Recent immigrants, insufficient family income, low social support and poor marital relationship are risk factors that can contribute to PPD over the 6 weeks following childbirth. There’s an urgent need for healthcare providers to take a more active role in engaging immigrant women in their psychological needs. Social support and culturally tailored programs should also be developed to prevent or alleviate depressive symptoms.

## Data Availability

The datasets used and/or analyzed during the current study are available from the corresponding author on reasonable request.

## Abbreviations

EPDS: Edinburgh Postnatal Depression Scale
PPD: postpartum depression
